# Impact of point-of-care ultrasound use on patient referral decisions in rural Kenya: a mixed methods study

**DOI:** 10.1186/s12913-024-10673-1

**Published:** 2024-02-15

**Authors:** Grace W. Wanjiku, Gregory Bell, Sonja Kapadia, Benjamin W. Wachira

**Affiliations:** 1 Department of Emergency Medicine, The Warren Alpert Medical School of Brown University, 55 Claverick Street Suite 101, Providence, RI 02903 USA; 2 Department of Emergency Medicine, Carver College of Medicine, University of Iowa, Iowa City, IA USA; 3 The Warren Alpert Medical School of Brown University, Providence, RI USA; 4 Accident and Emergency Department, The Aga Khan University, Nairobi, Kenya

**Keywords:** Point-of-care-ultrasound (POCUS), Kenya, Rural medicine, Referral

## Abstract

**Background:**

Point-of-care ultrasound (POCUS) is recognized as a key imaging modality to bridge the diagnostic imaging gap in Low- and Middle-Income Countries (LMICs). POCUS use has been shown to impact patient management decisions including referral for specialist care. This study explored the impact of POCUS use on referral decisions among trained healthcare providers working in primary rural and peri-urban health facilities in Kenya.

**Methods:**

A concurrent mixed methods approach was used, including a locally developed survey (*N* = 38) and semi-structured interviews of POCUS trained healthcare providers (*N* = 12). Data from the survey was descriptively analyzed and interviews were evaluated through the framework matrix method.

**Results:**

Survey results of in-facility access to Xray, Ultrasonography, CT scan and MRI were 49%, 33%, 3% and 0% respectively. Only 54% of the facilities where trainees worked had the capacity to perform cesarean sections, and 38% could perform general surgery. Through a combined inductive and deductive evaluation of interview data, we found that the emerging themes could be organized through the framework of the six domains of healthcare quality as described by the Institute of Medicine: Providers reported that POCUS use allowed them to make referral decisions which were timely, safe, effective, efficient, equitable and patient-centered. Challenges included machine breakdown, poor image quality, practice isolation, lack of institutional support and insufficient feedback on the condition of patients after referral.

**Conclusion:**

This study highlighted that in the setting of limited imaging and surgical capacity, POCUS use by trained providers in Kenyan primary health facilities has the potential to improve the patient referral process and to promote key dimensions of healthcare quality. Therefore, there is a need to expand POCUS training programs and to develop context specific POCUS referral algorithms.

**Supplementary Information:**

The online version contains supplementary material available at 10.1186/s12913-024-10673-1.

## Background

The use of point-of-care ultrasonography (POCUS) has emerged as a key imaging modality in developing countries [1]. This involves the use of ultrasound at the patient’s bedside by a clinician to answer a specific clinical question [2]. The utility of ultrasound has increased as the technology evolves to be more compact, portable, cost-effective and versatile. The World Health Organization (WHO) estimates that approximately two thirds of the world lacks access to diagnostic imaging and has recommended the use of POCUS to bridge this imaging disparity [3, 4].

Studies from LMICs illustrate that the use of POCUS leads to significant changes in the diagnosis and management of patients [5–11]. POCUS findings may trigger referral if higher-level or specialized care is indicated. Conversely, findings may suggest that care at the local, lower-level hospital is adequate. A POCUS training program was developed for healthcare providers working in primary care facilities in rural Kenya [12, 13]. The program was coupled with ultrasound donation and scanning was provided free or at a minimal fee to the patient. The program started in 2015, and a total of 150 healthcare providers of different cadres (physicians, mid-level providers, nurses and radiographers) at 60 facilities were trained.

Most of the facilities in which the Kenyan POCUS trainees work are small, isolated and under-resourced. The majority of their patients live in extreme poverty, defined by the world bank as earning $1.90 or less per day [14]. POCUS use can help determine if patients’ already limited resources need to be mobilized to obtain care at a referral center. Similarly, identifying patients that can be safely managed at the primary facility or safely discharged has significant cost saving implications.

A mixed methods cross-sectional study on the state of the healthcare referral system in Kenya revealed several challenges and gaps that could be addressed with POCUS [15]. Many patients are referred out of rural primary facilities based on perceived disease severity, but without definite diagnoses. This problem arises from lack of diagnostic equipment and leads to numerous inappropriate referrals. Further, lack of money for transportation and for diagnostic studies leads many patients to not complete their referrals.

The goal of this study was to use a mixed methods approach to understand the context in which the POCUS trainees work and how the use of POCUS impacts their decisions on whether to refer a patient out or manage them at their facility.

## Methods

A mixed methods approach was used to evaluate the resources available in the healthcare facilities where POCUS trainees work and the utility of POCUS use on their patient referral decisions.

### Study population

The target population was made up of Medical Officers (graduates from medical school), Clinical Officers (healthcare providers with a three-year diploma in clinical practice), Nurses and Radiographers that had been previously trained on POCUS use. Trained providers working in Level 6 national referral hospitals were excluded since they work at the highest level of the referral process.

### Sampling

The survey was deployed electronically to the email listserv of all the 150 healthcare providers who had received prior POCUS training through our program. The sampling approach for the qualitative arm of the study was based on four axes that we believe have a significant impact on patient management. These are professional background (Medical Officer, Clinical Officer, Nurse or Radiographer), level of facility in which they work, years of clinical practice, extent of POCUS training and POCUS clinical use. We selected a broad range of respondents who closely represented the healthcare workforce that is found in Kenyan rural and semi-urban healthcare facilities. Interviewees were recruited during a POCUS refresher /re-training course in the capital city Nairobi in Sept 2017, and during in-facility evaluations in January 2018. Interviewees were eligible even if they had participated in the online survey.

### Data collection

A structured survey that was developed locally following a literature review and local POCUS expert feedback (Appendix A) was used to collect quantitative data. The survey sought to collect demographic information, cadre of clinical training, years of clinical practice, healthcare facility level and resources available. It also included the number of POCUS training and refresher/re-training sessions, frequency of POCUS use and the POCUS modalities used. Finally, it evaluated how frequently the use of a specific modality triggers patient referral, and details of the referral process itself. The questionnaire and an associated consent form were deployed online using Qualtrics software and emailed to all eligible participants.

Interviews were conducted by three physicians who had received masters level training in emergency medicine including specific training on research methods and point-of-care ultrasound (GW, GB, BW). The interviewers used a semi-structured interview guide (Appendix B) that covered similar points as the questionnaire but included probing questions asking for specific cases in which the use of POCUS changed the referral plan. The interview also examined how POCUS changed day-to-day patient care, including challenges or opportunities it created. Providers who agreed to be interviewed signed consent forms or recorded their consent, and the interviews were recorded. Interviews were conducted in English which is one of the national languages in Kenya and is spoken by all healthcare providers who have gone through the formal training system.

### Data analysis

Data from the survey was analyzed using Excel. Descriptive statistics were used to assess responder demographics, professional cadre, years of clinical practice, level of healthcare facility, duration and frequency of POCUS practice. We also analyzed the resources available at the POCUS trainees’ facilities and which POCUS modalities were performed more frequently.

For qualitative analysis, the framework matrix method as described by Gale et al. (2013) was used for content and thematic analysis [16]. Recorded interviews were transcribed by a professional transcriber. Members of the study team read all the transcripts to familiarize themselves with the interviews. Two members of the research team (GW and SK) re-read the transcripts, performed open coding and independently generated an initial set of codes. The study team then met to discuss the codes, grouped them into categories and created an initial codebook. Each study member (GW, GB and SK) then re-read 3–4 transcripts using the initial codebook to check for any necessary additions and edits. The team met again to generate the final codebook.

A framework matrix was created on excel using the final codebook. A tab was created for each category, and the codes were placed on the columns. A row was added for every participant interview. The study team re-read the transcripts and performed indexing using the finalized codebook. Relevant and illustrative quotes from the interviews were summarized on the cells corresponding to the relevant code (Appendix C).

### Ethical considerations

This study was granted exemption by the Yale University Institutional Review Board under the category of: Research involving interviews, surveys, educational tests or observation of public behavior in which participant interaction includes providing a response to a non-physically invasive stimulus or behavioral activities commonly performed outside the research context (IRB protocol # 1,603,017,416). All procedures were performed in accordance with relevant guidelines.

## Results

### Characteristics of survey respondents

A total of 38 respondents completed the online survey. Most were male (*n* = 28, 73.7%), identified as clinical officers (*n* = 19, 50%), and worked at sub-county hospitals (*n* = 16, 42.1%). Most of the respondents reported 0–4 years of clinical practice (*n* = 18, 47.4%), and had 1 prior POCUS training session (*n* = 18, 47.4%). The characteristics of the respondents are shown on Table 1.

**Table 1:** Characteristics of survey respondents (*N* = 38)

Characteristics		N (%)
Gender	Male	28 (73.7)
	Female	10 (26.3)
Clinical Designation	Clinical Officer	19 (50.0)
	Nurse	12 (31.6)
	Medical Officer	5 (13.1)
	Radiographer	2(5.3)
Years of Clinical Practice	0–4	18 (47.4)
	5–9	12 (31.6)
	≥ 10	8 (21.0)
Number of POCUS Training Sessions	One	18 (47.4)
	Two	11 (28.9)
	Three or More	9 (23.7)
Facility Type	County	2 (5.3)
	Sub-County	16 (42.1)
	Health Center	10 (26.3)
	Clinic	2 (5.3)
	Dispensary	3 (7.9)
	Other (non-designated)	5 (13.1)

### Resource availability

The survey sought to investigate the resources available to the POCUS trainees as this was a key determinant of their decision to manage a patient in their own facilities or to refer them out for any concerning POCUS findings. We looked at availability of different imaging modalities, surgical capacity and availability of inpatient wards. Table 2. Shows the percentage of respondents who reported the presence of the specified resource either at their facility or at a specified distance away.

**Table 2:** Diagnostic imaging, surgical and in-patient care capacity

	At facility	1-10 km away	11-20 km away	> 20 km away
Ultrasound (not POC)	33.3%	25.0%	22.2	19.4
X-ray	48.7%	21.6%	13.5	16.2
CT Scan	2.9%	8.6%	22.9	65.7
MRI	0.0%	5.7%	14.3	80.0
C-Section capacity	54.1%	13.5	16.2	16.2
General Surgery	37.8%	16.2	18.9	27.0
Maternity ward	91.9%	5.4	2.7	0.0
Surgical ward	36.1%	13.9	25.0	25.0
Adult medical ward	83.8%	10.8	2.7	2.7
Pediatric ward	80.6%	8.3	5.6	5.6

POC = Point-of-Care, Km = Kilometer

Among trainees who reported performing > 10 bedside ultrasounds over the past month, 2nd /3rd trimester ultrasound was the most frequently performed (41.9%) followed by first trimester ultrasound (36.7%) E-FAST (7.1%) and echocardiography (0%). Obstetric POCUS was the modality that frequently triggered patient referral, with low lying placenta / placenta previa identified as the diagnosis that led to the most frequent referrals.

### Characteristics of the interviewees

Interviews were conducted with 12 healthcare providers. Most were male (*n* = 7, 58.3%), identified as clinical officers (*n* = 7, 58.3%), and worked in sub-county hospitals (*n* = 7, 58.3%). Most of the interviewees reported 0–4 years of clinical practice (*n* = 6, 50%) and had 1 prior POCUS training session (*n* = 6, 50%). The characteristics of the Interviewees are shown on Table 3.

**Table 3:** Characteristics of the interviewees (*N* = 12)

Characteristics		N (%)
Gender	Male	7 (58.3)
	Female	5 (41.7)
Clinical Designation	Clinical Officer	7 (58.3)
	Nurse	2 (16.7)
	Medical Officer	2 (16.7)
	Radiographer	1 (8.3)
Years of Clinical Practice	0–4	6 (50.0)
	5–9	2 (16.7)
	≥ 10	4 (33.3)
Number of POCUS Training Sessions	One	6 (50.0)
	Two	5(41.7)
	Three or More	1 (8.3)
Facility Type	County	2 (16.7)
	Sub-County	7 (58.3)
	Health Center	3 (25.0)
	Clinic	0
	Dispensary	0

### Analytical framework

The analytical framework was developed through a combined inductive and deductive analysis. The initial process was inductive, where emerging themes were identified from the transcribed interviews. However, in grouping the emerging themes into categories, we found that the themes could be organized through the framework of the six domains of healthcare quality as described by the institute of medicine [17]. These domains include Safety, Timeliness, Effectiveness, Efficacy, Equitability and Patient- centeredness. Table 4 shows the categories and codes that form the thematic analytical framework.

**Table 4:** Analytical categories and codes based on participants interviews

Category	Code
Safety	Determine if patient can be safely managed at facility
	Lack of operating theatre / Surgeon
	Avoid misdiagnosis
	Decreased mortality
Timeliness	Less wasting of time
	Reduced delays
Effectiveness	Decreased diagnostic uncertainly
	Avoid over/ under treatment
Efficiency	Increased clinician level efficiency: work faster, make decisions faster
	Increased facility level efficiency
	Increased clinician confidence and self-efficacy
Equitability	Reduced cost of care/cost saving
	Improved access for low SES status patients
Patient-Centeredness	Patient reassurance
	POCUS brings care to patient particularly the very sick/injured
	Patient appreciation of POCUS
	Community appreciation of POCUS

### Safety

Providers reported that POCUS use improved their ability to assess safety of labor and delivery and to evaluate life-threatening conditions such as ectopic pregnancy. They recognized POCUS as instrumental in determining if resulting diagnoses were within their capacity to manage. Of note in the survey, 92% of the respondents said they had inpatient maternity wards, but only 54% of those had the capacity to perform cesarean sections. This raises significant safety issues whereby a pregnant woman may be admitted to a facility that has no capacity to perform a cesarean section if needed.

*“It (POCUS) helps us in fact because if the baby is not in the right position and we don’t have a theater, definitely we will refer that patient for further management.” (Interviewee #7)*.

*“Actually, it is quite a lot in terms of making diagnosis especially in obstetric care. Like if you want to know if the patient is stable in your facility and she can deliver in your facility, you go through the ultrasound to ensure the patient, the precision is okay. We ensure even the fetus is okay, the fetal heart rate and we are able now to admit the patient. If we see any abnormalities, we refer very fast to the nearby facility because at the moment we do not have a theater in the facility.” (Interviewee # 9)*.

This was also true for surgical decision-making. Many interviewees reported limited general surgery capacity, leading to frequent referrals. In the survey, only 38% of the respondents had surgical capacity. Cases triggering surgical referral included fluid collection at the spleno-renal recess, hemothorax, and intra-abdominal fluid collection.

*“And trauma cases also, it’s very important because you might delay the patient. Especially if there’s hemothorax or internal bleeding. The more you stay with the patient, the more you might lose the patient. So, ultrasound has really helped us in terms of the referral system to work faster, to make decisions very fast.” (Interviewee #9)*.

Diagnostic imaging was significantly limited within the facilities where the interviewees worked. None of the interviewees had access to CT scan or MRI, X-ray services were inadequate, and patients had to wait in line for a long time. Some of the facilities had radiographers capable of performing ultrasound, but their hours were restricted.

*“A patient came with lower abdominal pains, spot(ing) maybe. We have a resident sonographer but he was not in, so I had to do the point-of-care ultrasound and we found that there is free fluid in the pelvis…. We had to refer outright because we don’t have a theater in our own facility.” (Interviewee #11)*.

### Timeliness

Almost all of the interviewed providers discussed the time-saving benefits of POCUS use for the patient, providers and the referral process as a whole. They emphasized the benefits of having a diagnosis to direct care and expedite transport to an appropriate facility.

*“But at least before I referred, I established what was the problem so at least when I’m referring, I’m referring directly to a specific health facility where he or she (can be) dealt with a lot faster.” (Interviewee #11)*.

Many described the traditional referral process as slow, requiring significant back-and-forth between medical officers, nurses, radiologists, and often consuming an entire day or more. These delays can contribute to patient morbidity and mortality.

*“Without the ultrasound, I could first send her to a radiologist, who could make a diagnosis, have an imaging done, come back with the result, then from the result, we refer. So, there is going back and forth.” (Interviewee #3)*.

*“So how much time would it have taken for the right diagnosis to be made?” (Interviewer)*.

*“That could take roughly a whole day… Even two, depending on the readiness of the patient too, because sometimes financially they are not able, so they will tell you “Let me first go get finances”. It can take even a whole month. Sometimes you even lose the patient.” (Interviewee #3)*.

Also highlighted were the financial difficulties that arise when patients are sent to a referral hospital without a specific diagnosis. Not only do they incur the cost of transportation, but they also have to wait in line and pay for medical consultation, wait at the cashier’s desk to submit payment for recommended tests then wait for diagnostic testing. This process is often labor intensive and consumes significant time.

*“I think it assists the referral process. Because you know when you send a patient, let’s say to MTRH, (a referral hospital) to have investigations done, a lot of time is spent waiting because they have to queue for this, queue for the other. Queue for finances, yeah, so it cuts that time.” (Interviewee #4)*.

### Effectiveness

Providers indicated that prior to POCUS use, their only tools were patient history and physical exam, resulting in increased referrals due to diagnostic uncertainty.

*“[Without POCUS], I would have done it in my own way, clinically. Using physical examination, touching the patient, examining and continue referring.” (Interviewee #6)*.

Thus, POCUS use decreases their diagnostic uncertainty, leads to changes in the patient management plan and allows them to better allocate resources and intervene earlier.

*“Yeah exactly, we were thinking of lots of things because in both cases the mothers were reporting that there was no fetal movement like they are used to. So, they were thinking that maybe they have lost their baby, and maybe they were having an abortion. We were in a mix up but at least when we did the ultrasound, we were able to tell that the baby was okay. To confirm.” (Interviewee #10)*.

In addition, providers reported that POCUS use decreased over or under-diagnosis and led to more appropriate referrals.

*“And again, it has reduced our referral - we used to refer a lot of patients for ultrasound. Nowadays we don’t refer, and it’s good we have been making the right diagnosis, and at least confirming the viability of the baby.” (Interviewee #10)*.

### Efficiency

Providers commented that POCUS makes them more efficient, enabling them to evaluate patients faster, and to expedite referrals when needed.

*“The fact that it was revealed was internal bleeding, of course that one will put people on toes. This is not a light matter. It quickened or it helped people to understand that this is a real emergency. The service or the attention changed pace.” (Interviewee #8)*.

POCUS use prompted providers to re-organize clinical operations to better optimize patient care.

*“The only challenge is that we have a lot of people we see during the day so we have to look for one day whereby all the antenatal cases are put then we check all the ladies and then we write what we have seen in the ultrasound so that it can make it easier for the midwives to know if they’ll continue with the care or if they don’t and to answer the question that made them ask for the ultrasound. So we choose one day in a month to do all that or two days, depending on the number that have been booked.” (Interviewee #7)*.

POCUS trained healthcare workers were able to make immediate triage decisions, even when a trained sonographer was not available.

*“The difference, we would have maybe waited for the sonographer to come because it was in the evening. Or maybe the patient would have waited until the following day, or we would have referred the patient altogether…without a diagnosis.” (Interviewee #11)*.

POCUS resulted in increased confidence and self-efficacy among practitioners.

*“Actually, my diagnosis now is perfect, also confidence in my work, even confidence from my staff. They know I’m doing ultrasound. Wanaambia wagonjwa tuna daktari anaweza…Kupima, anaweza kuangalia mtoto venye ako ndani. (They are telling patients that we have a doctor who can see and evaluate the baby inside) So I feel it has helped me a lot.” (Interviewee #9)*.

### Equitability

Multiple providers stated that POCUS is a free resource for patients at their facilities. This contributes to more equitable access to diagnostic studies. Patients are able to save on resources that would have been spent if POCUS was not available and incorrect diagnoses were made. Costs of misdiagnosis and inappropriate referrals include ambulance use, time and expense of nurses attending patient transport, and cost of consultation and diagnostic services at the referral hospital.

*“Yeah because our point-of-care, we don’t charge anything. We just do it. And it helps us to reduce their cost. Because once we send them outside, they have to pay.” (Interviewee #3)*.

*“With ultrasound care we are able to use even the NHIF (National Hospital Insurance Fund- low-cost national insurance) so it’s really helping patients.” (Interviewee #12)*.

*“It saves money because in our hospital, we could not use money for transportation with the ambulance, also the patient would not travel all that far, spending the time and money, money they will spend in the other hospital, which they would have used in their homes, their children, yeah.” (Interviewee #6)*.

### Patient-centeredness

Providers felt that POCUS use allowed them to provide care that was responsive to patients’ needs and values. For example, one provider reported that POCUS use allowed them to relieve patient distress and to provide reassurance in ways that they were not able to confidently do before.

*“Even these kinds of patients who come are feeling a lot of pain. You know in their mind they feel like… could it be that something is ruptured inside? But then after you do the E-FAST (Extended Focused Assessment with Sonography for Trauma) and you find that there is no internal bleeding. There is no pneumothorax. There is no hemothorax. You get? Somebody relaxes. It reassures the patient. Actually, that one is good for any sick person, the reassurance bit of it. So you find, had we not had this machine, how can you be sure or how can you be confident to tell the patient that all is well?” (Interviewee #8)*.

Another benefit was the POCUS machine portability, allowing providers to bring the machine to the patient.

*“There are some patients, maybe they have involved themselves in a serious accident and they cannot be moved in an easy way. The portable ultrasound helps you to go and just perform it right at the bedside… It is so easy to just come and do the scan at the bed there. I see it has really helped. It has really helped. Plus, the fact that it is portable. You charge it, then you move around with it.” (Interviewee #8)*.

POCUS use was associated with increased community appreciation. Providers reported that patients responded positively and were more confident in the care that they received.

*“Our community is really welcoming of ultrasound. They are very much in need of ultrasound” (Interviewee #6)*.

One provider indicated that word-of-mouth advertising about POCUS increased patient volumes (mostly in prenatal clinics) and that patients asked specifically for an ultrasound.

*“They know I’m doing an ultrasound. Wanaambiwa wagonjwa tuna daktari anaweza…Kupima, anaweza kuangalia mtoto venye ako ndani. (Patients are told that there is a doctor who can…evaluate and check how the fetus is doing inside) So I feel it has helped me a lot.” (Interviewee #9)*.

*“And then (they) come to disturb you, “I need an ultrasound.” (Interviewee #1)*.

### Challenges of POCUS use

Trainees expressed several challenges related to POCUS use. Some reported ultrasound machine related difficulties such as machine breakdown and poor image quality. Others reported lack of institutional support and opposition from other clinical staff since POCUS use was not standard of care. The lack of protected time to attend POCUS training and the fact that facilities were very busy prevented trainees from fully incorporating POCUS into their daily work. Practice isolation also came up as a theme, where many trainees did not have access to consultants to discuss concerning or indeterminate POCUS findings. Future work will explore these challenges in depth.

### Referral process

Once the decision is made to refer the patient out of the facility, the providers described the process and the resource mobilization required.

*“What generally we do is that we have to inform the patient verbally that our findings are this and this, we are referring you to a specialist who we do not have in our facility, you are going to a specific facility…and then we write a formal note to refer that patient, which we had to escort with the nurse because there must always be a nurse to go with the patient to wherever we are referring to. And then, we have to call that referral facility so that they have to receive this patient who is coming.” (Interviewee #3)*.

Our interviewees described the similar process of writing a formal referral note and calling the receiving facility. In the survey, 94% of the respondents reported that they have a formal referral protocol. The interviewees reported that in the best of circumstances they use an ambulance staffed by one of their nurses to transport the patient. Challenges include patients’ inability to pay for ambulance transport and frequent lack of fuel. One facility indicated that they have had to borrow an ambulance from a nearby (larger) county hospital. In the survey, 26% of the respondents reported that they always, 29% often, 40% sometimes and 5% never used an ambulance for patient transfers. The requirement to use a staff nurse to transport a patient, mostly due to lack of trained emergency medical technicians (EMTs) or paramedics, exacerbates staff shortages at primary facilities. Distance to the closest referral facility also affected the degree of resource mobilization and choices available to complete a referral. A provider whose facility is in a peri-urban area indicated that they have several higher-level facilities close by, and therefore had more options for transport e.g. taxi that are not financially prohibitive. On the other hand, a more remote facility sends patients approximately 100 km to the closest referral hospital, necessitating the use of an ambulance and a staff nurse in cases with high risk for deterioration. The stability of the patient was also discussed as a factor determining resource mobilization. Other providers brought up a strike that had taken place in government / public referral hospitals, causing patients to be referred further out to private referral hospitals that were more expensive. Thus, we see the impact of patients’ stability, ability to pay, distance to the closest referral hospital and potential staffing deficiencies at referral facilities affecting the choice of transportation and monitoring along the way. The significant degree of resource mobilization required to complete a referral underscored the importance of using POCUS, and its cost saving potential if inappropriate referrals can be avoided.

The providers reported that once the patient is referred out, they rarely get an update on their condition. One community hospital had a thorough follow up process, but others only had sporadic updates on the patients’ condition. Whenever available, patient follow up particularly where the outcome was positively influenced by POCUS, helped to further reinforce and encourage POCUS practice.

*“The new case I’ve faced there is a young man who got an accident. Upon doing a FAST scan, we found that there is a fluid collection at the spleno-renal recess. And having that history in mind then that one automatically qualified to be internal bleeding. So, because we do not have people who can perform such an operation there and I found the medical officer was not around and so, he was referred to the county hospital and he was done an operation. The spleen had ruptured. He was done a spleen… I don’t.” (Interviewee #8)*.

*“Splenectomy?” (Interviewer)*

*“Yeah. A few months later he came back for just checkup then he reminded me that he was once there and I did the scan. He was very happy. And I’m also even more happy than him.” (Interviewee #8)*.

## Discussion

To examine how POCUS use affects their referral decisions, healthcare providers focused on provider and patient outcomes. The themes that emerged from the qualitative evaluation were clustered around and can be contextualized through the six domains of healthcare quality as defined by the institute of medicine [17]. These emerging themes were further supported by the survey data that illustrated the context in which the healthcare providers work and the environment in which the new technology (POCUS) has been introduced.

The most recurrent of these themes was ensuring patient **safety** in the face of limited diagnostic imaging and surgical capacity. Other studies in resource-limited settings support POCUS use to screen patients with potentially high-risk pregnancies [18, 19]. A study in the Philippines showed that the performance of focused obstetrics by trained community healthcare workers may have possibly averted 6.3% maternal deaths and 14.6% neonatal deaths at the time of delivery [20].

**Timeliness** came up as a theme; providers emphasized that the usual process of determining patient disposition is long in the context of high patient volumes and limited resources. Studies have shown that non-experts in low resource settings can be trained to use POCUS as a **screening tool** and to provide **early linkages to care** for patients with rheumatologic heart disease and other structural heart diseases [21–28].

The use of POCUS was associated with increased **effectiveness** in that providers were able to make diagnoses that they were not able to confidently make before, bolstering feelings of their own self-efficacy. Previous studies have shown that the use of POCUS in low-resource settings has led to increased diagnostic accuracy and resulted in new treatment interventions that were not previously considered [29, 30].

POCUS trained providers felt that they were more **efficient**, both on a personal and facility level. The ability to provide POCUS to patients at no cost or minimal cost allowed providers to provide **equitable and cost-effective** care. The study by Marin-Gomez FX et.al. shows that POCUS use not only saves time and money, but also reduces fuel use and emission of air pollutants [31].

The ability to use ultrasound at the patients’ bedside engendered **patient-centered care**, contributed to patient reassurance and led to increased community appreciation of the care received. The use of POCUS in primary or general care has been associated with enhanced patient experience, increased reassurance and patients’ trust that they have been thoroughly examined [32]. Healthcare providers reported that POCUS use was associated with increased healthcare utilization. Previous studies have demonstrated that the use of POCUS boosts antenatal care attendance [33, 34].

In interrogating the referral process, we found that the link between rural providers and the referral hospitals was largely one sided, and that rural providers don’t always get feedback on the condition of the patient post-referral. Further, rural providers did not always get feedback as to whether their POCUS results and risk stratification of patients made a difference in how the patients were triaged and managed at the referral hospital. Thus, more needs to be done to improve the continuum of care between rural facilities and referral hospitals with attention to POCUS use [19].

This study has some limitations and potential sources of bias. Firstly, the number of survey respondents was low, with only a quarter of all eligible participants returning a completed survey. Despite the small number of survey respondents, they represented various regions in the country, and the sample was representative in terms of cadre and level of facility from which all our POCUS trainees were drawn. The survey and interviewee samples were also matched in terms of cadre, facility level, years of clinical practice and number of previous POCUS training sessions. These samples are representative of the overall rural POCUS training cohort.

Another limitation is that in the semi-structured interviews providers gave their recollection of memorable cases whose management was altered by POCUS use. We found that they reported cases that they believe were positively impacted by POCUS, but not instances where POCUS use may have harmed a patient, especially if a POCUS result was mis-interpreted. POCUS trainees undergo regular quality assurance and evaluations every 3 to 4 months, but the possibility of ultrasound image misinterpretation cannot be completely ruled out. Furthermore, since they do not routinely get feedback on the health status of the patients they referred out, trainees might not be aware of times that they might have made a wrong diagnosis or an inappropriate referral. Focus groups were not conducted, and it is possible that they could have added more richness to the data. However, the emerging themes were recurrent in the interviews and were additionally supported by survey responses. Lastly, within our program, POCUS exams are performed for free or at a minimal fee to patients. In the future when POCUS becomes standard of care, the services may be billed as a procedure as is currently the case in high income settings [35]. While not as costly as a radiology performed study, this might create additional barriers for patient care.

## Conclusion

This study showed that POCUS use plays a key role in assisting non-radiologist healthcare providers in rural, under-resourced facilities to make patient management decisions that involve referral to larger facilities. The study illustrated the significant diagnostic imaging and surgical capacity limitations in the facilities where POCUS trained providers work. In this context, POCUS use can improve the diagnostic and referral process and as a result improve key dimensions of healthcare quality. Further work could focus on a closer evaluation of patient cases managed with POCUS to determine which cases are routinely referred versus managed at the rural / peri-urban facilities. This process could lead to the development of POCUS referral algorithms that are agreed upon between the rural and referral hospitals, and as a result improve the care continuum between them.

### Electronic supplementary material

Below is the link to the electronic supplementary material.

Supplementary Material 1

Supplementary Material 2

Supplementary Material 3

## Data Availability

The datasets used and analyzed during the current study are available from the corresponding author on reasonable request.

## References

[1] Sippel S, Muruganandan K, Levine A, Shah S (2011). Review article: use of ultrasound in the developing world. Int J Emerg Med.

[2] Moore CL, Copel JA (2011). Point-of-care ultrasonography. N Engl J Med.

[3] Medical devices.: Managing the Mismatch. [cited 2022 Jan 31]. Available from: https://www.who.int/publications-detail-redirect/medical-devices-managing-the-mismatch.

[4] Reynolds TA, Sawe H, Rubiano AM, Shin SD, Wallis L, Mock CN et al. Strengthening Health Systems to Provide Emergency Care. In: Jamison DT, Gelband H, Horton S, Jha P, Laxminarayan R, Mock CN, editors. Disease Control Priorities: Improving Health and Reducing Poverty. 3rd ed. Washington (DC): The International Bank for Reconstruction and Development / The World Bank; 2017 [cited 2022 Jan 31]. Available from: http://www.ncbi.nlm.nih.gov/books/NBK525279/.

[5] Shah SP, Epino H, Bukhman G, Umulisa I, Dushimiyimana JMV, Reichman A (2009). Impact of the introduction of ultrasound services in a limited resource setting: rural Rwanda 2008. BMC Int Health Hum Rights.

[6] Kotlyar S, Moore CL (2008). Assessing the utility of ultrasound in Liberia. J Emerg Trauma Shock.

[7] Kobal SL, Lee SS, Willner R, Aguilar Vargas FE, Luo H, Watanabe C (2004). Hand-carried cardiac ultrasound enhances healthcare delivery in developing countries. Am J Cardiol.

[8] Shorter M, Macias DJ (2012). Portable handheld ultrasound in austere environments: use in the Haiti disaster. Prehosp Disaster Med.

[9] Kolbe N, Killu K, Coba V, Neri L, Garcia KM, McCulloch M (2015). Point of care ultrasound (POCUS) telemedicine project in rural Nicaragua and its impact on patient management. J Ultrasound.

[10] Kimberly HH, Murray A, Mennicke M, Liteplo A, Lew J, Bohan JS (2010). Focused maternal ultrasound by midwives in rural Zambia. Ultrasound Med Biol.

[11] Blaivas M, Kuhn W, Reynolds B, Brannam L (2005). Change in differential diagnosis and patient management with the use of portable ultrasound in a remote setting. Wilderness Environ Med.

[12] Bell G, Wachira B, Denning G (2016). A pilot training program for point-of-care ultrasound in Kenya. Afr J Emerg Med.

[13] Wanjiku GW, Bell G, Wachira B (2018). Assessing a novel point-of-care ultrasound training program for rural healthcare providers in Kenya. BMC Health Serv Res.

[14] World Bank. [cited 2022 Jan 31]. FAQs: Global Poverty Line Update. Available from: https://www.worldbank.org/en/topic/poverty/brief/global-poverty-line-faq.

[15] Gitonga C, Jennrich. Lynn. Kenya Ministry of Health: The state of the Health Referral System in Kenya: Results from a baseline study on the functionality of the health referral system in eight counties. 2013.

[16] Gale NK, Heath G, Cameron E, Rashid S, Redwood S (2013). Using the framework method for the analysis of qualitative data in multi-disciplinary health research. BMC Med Res Methodol.

[17] America I, of M (US.) C on Q of HC in. Improving the 21st-century Health Care System. Crossing the Quality Chasm: A New Health System for the 21st Century. National Academies Press (US); 2001 [cited 2022 Jan 31]. Available from: https://www.ncbi.nlm.nih.gov/books/NBK222265/.

[18] Vyas A, Moran K, Livingston J, Gonzales S, Torres M, Duffens A (2018). Feasibility study of minimally trained medical students using the rural obstetrical ultrasound triage exam (ROUTE) in rural Panama. World J Emerg Med.

[19] Swanson JO, Kawooya MG, Swanson DL, Hippe DS, Dungu-Matovu P, Nathan R (2014). The diagnostic impact of limited, screening obstetric ultrasound when performed by midwives in rural Uganda. J Perinatol.

[20] Dalmacion GV, Reyles RT, Habana AE, Cruz LMV, Chua MC, Ngo AT (2018). Handheld ultrasound to avert maternal and neonatal deaths in 2 regions of the Philippines: an iBuntis® intervention study. BMC Pregnancy Childbirth.

[21] Beaton A, Aliku T, Okello E, Lubega S, McCarter R, Lwabi P (2014). The utility of handheld echocardiography for early diagnosis of rheumatic heart disease. J Am Soc Echocardiogr.

[22] Colquhoun SM, Carapetis JR, Kado JH, Reeves BM, Remenyi B, May W (2013). Pilot study of nurse-led rheumatic heart disease echocardiography screening in Fiji–a novel approach in a resource-poor setting. Cardiol Young.

[23] Lu JC, Sable C, Ensing GJ, Webb C, Scheel J, Aliku T (2015). Simplified rheumatic heart disease screening criteria for handheld echocardiography. J Am Soc Echocardiogr.

[24] Mirabel M, Bacquelin R, Tafflet M, Robillard C, Huon B, Corsenac P (2015). Screening for rheumatic heart disease: evaluation of a focused cardiac ultrasound approach. Circ Cardiovasc Imaging.

[25] Ploutz M, Lu JC, Scheel J, Webb C, Ensing GJ, Aliku T (2016). Handheld echocardiographic screening for rheumatic heart disease by non-experts. Heart.

[26] Kirkpatrick JN, Nguyen HTT, Doan LD, Le TT, Thai SP, Adams D (2018). Focused Cardiac Ultrasound by nurses in Rural Vietnam. J Am Soc Echocardiogr.

[27] Ansa VO, Odigwe CO, Agbulu RO, Odudu-Umoh I, Uhegbu V, Ekripko U (2013). The clinical utility of echocardiography as a cardiological diagnostic tool in poor resource settings. Niger J Clin Pract.

[28] Bhavnani SP, Sola S, Adams D, Venkateshvaran A, Dash PK, Sengupta PP (2018). A Randomized Trial of Pocket-Echocardiography Integrated Mobile Health Device Assessments in Modern Structural Heart Disease clinics. JACC Cardiovasc Imaging.

[29] Lamorte A, Boero E, Crida P, Conteh AR, Foletti M, Narcisi P (2016). The Sierra Leone Ultrasound Rainbow4Africa Project (SLURP): an observational study of ultrasound effectiveness in developing countries. Crit Ultrasound J.

[30] Stachura M, Landes M, Aklilu F, Venugopal R, Hunchak C, Berman S (2017). Evaluation of a point-of-care ultrasound scan list in a resource-limited emergency centre in Addis Ababa Ethiopia. Afr J Emerg Med.

[31] Marín-Gomez FX, Mendioroz Peña J, Canal Casals V, Romero Mendez M, Darnés Surroca A, Nieto Maclino A (2020). Environmental and patient impact of applying a point-of-care Ultrasound Model in Primary Care: Rural vs. Urban centres. Int J Environ Res Public Health.

[32] Andersen CA, Brodersen J, Rudbæk TR, Jensen MB (2021). Patients’ experiences of the use of point-of-care ultrasound in general practice - a cross-sectional study. BMC Fam Pract.

[33] Amoah B, Anto EA, Osei PK, Pieterson K, Crimi A (2016). Boosting antenatal care attendance and number of hospital deliveries among pregnant women in rural communities: a community initiative in Ghana based on mobile phones applications and portable ultrasound scans. BMC Pregnancy Childbirth.

[34] Cherniak W, Anguyo G, Meaney C, Yuan Kong L, Malhame I, Pace R (2017). Effectiveness of advertising availability of prenatal ultrasound on uptake of antenatal care in rural Uganda: a cluster randomized trial. PLoS ONE.

[35] Hughes D, Corrado MM, Mynatt I, Prats M, Royall NA, Boulger C (2020). Billing I-AIM: a novel framework for ultrasound billing. Ultrasound J.

