# Supplementary material for: Impact of point-of-care ultrasound use on patient referral decisions in rural Kenya: a mixed methods study
Source: BMC Health Serv Res. 2024 Feb 15;24:212. doi: 10.1186/s12913-024-10673-1 (PMC10870490; doi:10.1186/s12913-024-10673-1)
Supplement: Supplementary file 1 — Supplementary Material 1 [file 12913_2024_10673_MOESM1_ESM.pdf]

Appendix A: Online Questionnaire

Age ___________(Years) Sex ☐Male ☐Female

FACILITY INFO

1. How would you characterize the facility in which you work?

   ☐ Dispensary
   ☐ Clinic
   ☐ Health center
   ☐ Sub county hospital
   ☐ Other (specify)

2. What is the approximate number of people living in the area(s) served by your facility? (Catchment area) **[MEASURE evaluation, 2013. Adapted]**

   ☐ < 5000
   ☐ 5000-10,000
   ☐ 10001-15,000
   ☐ > 15000
   ☐ I don't know

3. How many patients does your facility serve everyday? (Approximate outpatient)
   ☐ 0-50
   ☐ 51-100
   ☐ 101-150
   ☐ 151-200
   ☐ >200

4. How many total inpatient beds does your facility have?
   ☐ 0
   ☐ 1-25
   ☐ 26-50
   ☐ 51-75
   ☐ 76-100
   ☐ > 100

5. How many of the following health care providers work at your facility (including yourself)

Medical officers	☐ 0	☐ 1	☐ 2	☐ 3	☐ 4	☐ 5 or more
Clinical officers	☐ 0	☐ 1	☐ 2	☐ 3	☐ 4	☐ 5 or more
Nurses	☐ 0	☐ 1	☐ 2	☐ 3	☐ 4	☐ 5 or more
Midwives	☐ 0	☐ 1	☐ 2	☐ 3	☐ 4	☐ 5 or more

6. Please indicate if you have the following services at your facility and if not, approximately how far away these services are located

**Service**	**At my facility**	**1-10 Km**	**11- 20Km**	**More than 20 km**
Formal ultrasound (not point of care)				
X-ray				
CT scan				
MRI				
OB surgery- Caesarean section				
General Surgery				
Maternity ward				
General surgery ward				
Adult Medical ward				
Pediatric ward				

POINT OF CARE ULTRASOUND USE

7. Your position

☐ Medical officer
☐ Clinical officer
☐ Nurse
☐ Radiographer/ ultrasonographer

8. Years you have been in clinical practice (exclude time spent in training)

☐ 0-4
☐ 5-9
☐ 10 or more

9. How many point of care ultrasound training sessions have you participated in? (include initial training and refreshers)

☐ 1
☐ 2
☐ 3
☐ 4
☐ 5

10. How many ultrasounds have you performed yourself over the past month?

☐ 0
☐ 1-5
☐ 6-10
☐ >10

11. How many of each ultrasound type do you estimate to have performed over the past month?

**Ultrasound Type**	**0**	**1-5**	**6-10**	**>10**
EFAST				
Echocardiography				
OB – 1st trimester				
OB -2nd and 3rd trimester				

12. In the past month, how often has the use of point of care ultrasound led you to send a patient to get the following?

	**Never**	**Sometimes**	**Often**	**Always**
A formal ultrasound by a radiographer or radiologist ?				
An formal echocardiogram				
An x-ray				
A CT scan				
An MRI				
A Caesarean section?				
A General surgical procedure				
Admission at your hospital or nearest referral facility				

13. Please indicate how often the different types of ultrasounds below have led you to send a patient for referral

**Ultrasound Type**	**Never**	**Sometimes**	**Often**	**Always**
EFAST				
Echocardiography				
OB – 1st trimester				
OB -2nd and 3rd trimester				

14. What diagnoses have you made by using point of care ultrasound that led you to send a patient for a referral?

**Diagnosis**	**0**	**1-5**	**6-10**	**>10**
Ectopic pregnancy				
Breech presentation				
Twins				
Low lying placenta/ previa				
Abnormal foetal heart beat				
Foetal demise				
Free fluid in the peritoneum				
Free fluid in the pleural space				
Free fluid in the pericardium				

15. Please indicate how often the different types of ultrasounds below have led you to AVOID referring a patient that you would have normally referred

**Ultrasound Type**	**Never**	**Sometimes**	**Often**	**Always**
EFAST				
Echocardiography				
OB – 1st trimester				
OB -2nd and 3rd trimester				

16. If you have a question/ or an unsure about an ultrasound finding, do you have a consultant at your facility to discuss your questions/ concerns with?

☐ Never
☐ Sometimes
☐ Often
☐ Always

17. If you have a consultant to help review your point of care ultrasounds, what is their specialty?

☐ Radiologist (medical school trained)
☐ Radiographer (not medical school trained)
☐ Surgeon
☐ Physician- general medicine practitioner
☐ Other (please specify)

REFFERAL PROCESS [MEASURE evaluation, 2013. Adapted]

18. How would you characterize the facility to which you refer most of your patients?

☐Sub county hospital
☐County hospital
☐National public referral hospital
☐Faith based referral hospital
☐Other (specify)

19. Does your facility have a formal referral protocol?

☐Yes ☐No

20. What method do you use to refer patients? Check all that apply

☐ Verbal (tell them where to go)
☐ Telephone referral
☐Give them a standard referral form
☐Blank slip of paper to write referral information
☐Escort patient
☐Other__________________

21. Is there a formal agreement between your facility and the receiving referral hospitals to which you send your patients?

☐Yes ☐No

22. How do most patients get to the referral facility to which they have been sent

**Transportation**	**Never**	**Sometimes**	**Often**	**Always**
Ambulance				
Personal car				
Taxi				
Matatu				
Boda Boda				
By foot				

23. For patients who are very sick how do they get to the referral facility?

**Transportation**	**Never**	**Sometimes**	**Often**	**Always**
Ambulance				
Personal car				
Taxi				
Matatu				
Boda Boda				
By foot				

24. Does your facility have a record keeping system to track patients who have been referred out?

☐Yes ☐No

25. Do you have a system to determine if the patient has completed the referral that you gave them

☐Yes ☐No

26. If yes, please specify by checking all that apply

☐ Verbal (tell them where to go)
☐ Telephone referral
☐Give them a standard referral form
☐Blank slip of paper to write referral information
☐Escort patient
☐Other__________________

COST CONSIDERATIONS

27. How much does a point of care OB/ GYN ultrasound cost at your facility

☐ 0- 500 Ksh
☐501-1000 Ksh
☐ 1001-1500
☐>1501 Ksh
☐I don't know

28. How much does an OB/ GYN ultrasound cost at the closest referral hospital?

☐ 0- 500 Ksh
☐ 501-1000 Ksh
☐ 1001-1500
☐ >1501 Ksh
☐ I don't know

29. How much does an OB/ GYN ultrasound cost at the closest private radiology facility?

☐ 0- 500 Ksh
☐ 501-1000 Ksh
☐ 1001-1500 Ksh
☐ >1501 Ksh
☐ I don't know
