# Supplementary material for: Impact of point-of-care ultrasound use on patient referral decisions in rural Kenya: a mixed methods study
Source: BMC Health Serv Res. 2024 Feb 15;24:212. doi: 10.1186/s12913-024-10673-1 (PMC10870490; doi:10.1186/s12913-024-10673-1)
Supplement: Supplementary file 2 — Supplementary Material 2 [file 12913_2024_10673_MOESM2_ESM.docx]

**Appendix B: Interview guide**

I’m doing this interview to get a better understanding your experience with using point of care ultrasound in your facility. In particular, I would like to know how using the ultrasound affects how you refer patients. Thank you for taking the time to share about your experience. I would like to record this for note-taking purposes: Is that okay with you? If at any time you want to turn the recording off we can do that.

I’ve prepared a set of questions to guide our conversation- but feel free to bring up any points I might have left off. If any question is unclear or makes you uncomfortable, let me know as well so we can re-direct the conversation. Are you ready to get started?

Can you tell me a little bit more about yourself and your health care training?

Probing questions

-Type of medical training

-How long ago the training was completed

-How long have they been at current facility?

When did you receive your point of care ultrasound training?

Probing questions

-Initial training

-In-facility evaluation

-Refresher course

-If they are already credentialed (completed the point of care ultrasound training process)

What has your experience been so far with the use of point of care ultrasound?

Probing questions

-How are you integrating point of care ultrasound in your daily work?

-How has ultrasound impacted your patient management in general?

-Is point of care ultrasound improving your practice or making it worse?

-Challenges in using a new technology

- Is there anything that point of care ultrasound has allowed you to do, that you were not able to do before you had it?

Can you tell me about a particular case in which point of care ultrasound caused you to refer a patient?

Probing question

-Specific ultrasound modality (FAST, Cardiac, Obstetric) that was used

-What diagnosis was made that triggered transfer

- Did you have an alternate diagnosis in mind?

Tell me more about how you made the referral

Probing questions

-What facility was the patient sent to?

-How far away was the referral facility?

-Mode of transport to that facility

-Clinical condition of the patient (stable? In extremis?)

--Documentation of the referral

Do you know what happened to the patient after you referred them?

Probing question

-Did they undergo a lifesaving procedure?

-Are there mechanisms that exist for providers to determine if a referral to another facility has been completed?

How would you have managed this case if you did not have an ultrasound?

Probing question

-Did you have a different diagnosis in mind?

-How much time would it have taken for the right diagnosis to be made?

Do you have a case in which the use of point of care ultrasound prevented you from referring a patient that you otherwise would have?

Probing question

-Did ultrasound use save the patient money and time?

In your experience does point of care ultrasound help the referral process or make it worse?

How can point of care ultrasound be used to improve the referral process?
